# Event and Comment

**Published:** 1920-04

**Authors:** 


### DENTAL REGISTER

THE MAGAZINE OF DENTISTRY

Vol. LXXIV APRIL, 1920 No. 4

### EVENT AND COMMENT

**Tooth Troubles** No dental trouble is more frequently discussed at the present time than that relating to the vitality of the pulp of the tooth, and no disease of this organ is more liable to serious consequences as related to special health or general systemic infection. We begin dental disturbances with the developing tooth and we almost think we never seem quite free from it, as long as there remains a single tooth in the jaws. We have studied the problem of dental health from every possible angle, embryologic, physiologic, pathologic, surgical and medicinal, to detailed oral hygiene. Nothing seems left but prosthetic restorations. We have gained somewhat in scientific knowledge and technical skill, but we have not simplified our treatment nor apparently made satisfactory gains; in fact, our problem in some respects seems more complicated than before. When Mayo said the next great health problem was to be a dental problem, he made a wiser prediction than he knew, and we doubt if the majority of dentists realize that the teeth are so intimately related with the entire body as to produce such general health disturbances as are now troubling us.

We shall need to learn at once, and well, that the anatomy and physiology of the jaws are not fractions of the human body to be studied, but intergers, otherwise we shall not be able to interpret correctly our disease phenomena. We have been active, and to some extent erratic, in ascribing many diseases to conditions which we have not been able to prove out, and to some degree we have asserted a course of diagnosis and have attempted a class of therapy to fit it.

In such confusion are we not likely to adopt erratic experimentation in place of holding firmly to the knowledge we have gained by long experience and scientific deductions, even though we may be accused of dogmatizing about some of our methods which we may think are too empirical for our age and knowledge? For instance, will it be wise for us to propose to destroy the pulps of all teeth that may be perilously irritated, or those in which the pulps have been eradicated, or those whose periapical regions are not seriously involved in infection? Is there no hope for the cure of the peridental abscess, and must we consign to the forceps all teeth which we may not believe, theoretically, will ultimately become sources of systemic infection?

This is not in accordance with our idea of progress and can not be conservative dentistry. It is our notion that, in spite of the fact that there is abundant evidence to substantiate the findings of our medical and dental diagnosticians, we are not yet in a position to overthrow the foundation upon which we have constructed our entire system of clinical practice. It is probably true that we have overreached good judgment in artistic technique in some cases, but it is more likely that we have fallen far short of adequate technical excellence, and that to this fact we must attribute most of our disastrous failures of ill-health. We shall, of course, have to use better judgment in the future in caring for the ravages caused by oral insanitation, but we see no reason why, with more knowledge and the additional care we are giving to oral hygiene, we may not hope for the elimination of systemic disease to a larger extent than seems probable at the present time. Let us, therefore, go on perfecting our dental technique, because we are likely to accomplish more in this way, and in the meantime we should enlist our scientific research men in an effort to strengthen biologically our natural health resources in so far as this is practicable.

